# Association between occlusal support and cognitive impairment in older Chinese adults: a community-based study

**DOI:** 10.3389/fnagi.2023.1146335

**Published:** 2023-04-17

**Authors:** Dongxin Da, Suyu Ge, Hao Zhang, Xiaoli Zeng, Yiwei Jiang, Jin Yu, Huning Wang, Wanqing Wu, Zhenxu Xiao, Xiaoniu Liang, Qianhua Zhao, Ding Ding, Ying Zhang

**Affiliations:** ^1^Department of Preventive Dentistry, Shanghai Stomatological Hospital and School of Stomatology, Fudan University, Shanghai, China; ^2^Shanghai Key Laboratory of Craniomaxillofacial Development and Diseases, Fudan University, Shanghai, China; ^3^Institute of Neurology, Huashan Hospital, Fudan University, Shanghai, China; ^4^National Center for Neurological Disorders, Huashan Hospital, Fudan University, Shanghai, China; ^5^National Clinical Research Center for Aging and Medicine, Huashan Hospital, Fudan University, Shanghai, China

**Keywords:** cognitive dysfunction, dental occlusion, mastication, mediation analysis, dental health survey

## Abstract

**Introduction:**

The loss of occlusal support due to tooth loss is associated with systemic diseases. However, there was little about the association between occlusal support and cognitive impairment. The cross-sectional study aimed to investigate their association.

**Methods:**

Cognitive function was assessed and diagnosed in 1,225 community-dwelling adults aged 60 years or older in Jing’an District, Shanghai. Participants were diagnosed with mild cognitive impairment (MCI) by Peterson’s criteria, or dementia, according to the Diagnostic and Statistical Manual of Mental Disorders, Fourth Edition. We determined the number of functional occlusal supporting areas according to Eichner classifications. We used multivariate logistic regression models to analyze the relationship between occlusal support and cognitive impairment and mediation effect models to analyze the mediation effect of age.

**Results:**

Six hundred sixty participants were diagnosed with cognitive impairment, averaging 79.92 years old. After adjusting age, sex, education level, cigarette smoking, alcohol drinking, cardiovascular disease, and diabetes, individuals with poor occlusal support had an OR of 3.674 (95%CI 1.141–11.829) for cognitive impairment compared to those with good occlusal support. Age mediated 66.53% of the association between the number of functional occlusal supporting areas and cognitive impairment.

**Discussion:**

In this study, cognitive impairment was significantly associated with the number of missing teeth, functional occlusal areas, and Eichner classifications with older community residents. Occlusal support should be a significant concern for people with cognitive impairment.

## 1. Introduction

With the population aging, cognitive impairment, including dementia and mild cognitive impairment (MCI), has become a public health focus (Jia et al., 2020). Dementia is a leading cause of disability and dependency in older adults and can disrupt the lives of patients, their careers, and their families. In addition, dementia imposes a heavy economic burden on society, with the cost of caring for people with dementia estimated to rise to $2 trillion annually (World Health Organization, 2019). In 2019, approximately 50 million people worldwide lived with dementia. There are nearly 10 million new cases yearly (Livingston et al., 2020), and China accounts for about a quarter of them. MCI refers to the early stages of cognitive decline and is generally considered the transitional stage between normal aging and dementia (Petersen, 2010; Nelson et al., 2021). Those patients who eventually develop dementia have minimal initial cognitive impairment. A diagnosis of MCI helps to treat these patients timely. *Via* a national cross-sectional study in China, Jia et al. (2020) estimated that the prevalence of dementia was 6.0%, with the prevalence of MCI 15.5%. Between 30 and 50% of patients worldwide developed dementia from MCI within 5 to 10 years (Liss et al., 2021). Patients with MCI have a certain chance of turning normal or developing into dementia. Ding et al. (2016) found the conversion rate of MCI to dementia was 6.0 per 100 person-years, while the reversion rate to cognitive normal was 7.8 per 100 person-years.

Risk factors for cognitive impairment include age, genetic factors, and lifestyle habits. Recent studies have found that occlusal support was also one of the risk factors for cognitive impairment (Miura et al., 2003; Ono et al., 2010; Mummolo et al., 2014; Weijenberg et al., 2019; Kim et al., 2020). Tooth loss caused by periodontal disease can lead to decreased masticatory function, malnutrition, and accelerated neurodegeneration in the brain due to lack of stimulation and increased cognitive impairment risk (Ono et al., 2010). Yamamoto et al. (2012) found few teeth and poor chewing were associated with dementia onset among 4,425 Japanese residents 65 years or older.

Mastication enables food to mix well with salivary enzymes and gastric acid, which facilitates food digestion and nutrient absorption and stimulates oral receptors and the nervous system. The functional occlusal supporting areas and the Eichner classifications are recognized globally as valid indicators of masticatory function (Ikebe et al., 2005, 2010b). Eichner developed the Eichner classification in the 1950s to measure occlusal support based on the functional occlusal supporting areas (Eichner, 1955, 1990; Ikebe et al., 2010b). Significant correlations of the Eichner index with tooth loss, masticatory function, and TMJ disorders have been proven by several studies (Ikebe et al., 2010a,b; Miura et al., 2010).

Due to the inconsistent results and the lack of studies on the mediation effect of age, our study aimed to investigate the association between occlusal support and cognitive impairment and the mediation effect of age among community-dwelling older adults, which was essential for further studies on the pathogenesis and prevention of cognitive impairment.

## 2. Materials and methods

### 2.1. Study population

From January 2019 to December 2019, community dwellers aged 60 years or older living in Jing’an District, Shanghai, were eligible to be recruited for oral examination and clinical interview.

Exclusion criteria were (1) lived in nursing homes or other institutions;(2) experienced mental deficiency or severe schizophrenia, according to their medical record or diagnosis from neurologists; or (3) had severe impairment of hearing, vision, or verbal such that could not accomplish the neuropsychological evaluation or oral examination. Recruitment details have been published elsewhere (Ding et al., 2015).

The Medical Ethics Committee of Huashan Hospital, affiliated with Fudan University, approved this study. All participants and/or their guardians have given written informed consent.

### 2.2. Oral health examination

According to the World Health Organization’s Basic Methods for Oral Health Surveys (5th edition) (World Health Organization, 2013), two dentists who got trained and passed the interrater reliability test examined the participants’ oral health. The Cohen’s kappa value for the dentists was 0.85, which achieved good agreement quality (Landis and Koch, 1977). The dentists examined all the participants’ teeth and occlusal contact and calculated the number of remaining teeth and functional occlusal areas.

The stage of periodontitis was determined according to the new classification of periodontal diseases in 2018 (Tonetti et al., 2018; Tonetti and Sanz, 2019). The occlusal support was classified using the number of functional occlusal supporting areas and the Eichner index. There were four functional occlusal supporting areas, namely the premolars and the molars on both sides. Each area was considered one functional occlusal area if occlusal contact existed. The participants were divided into three groups according to the number of functional occlusal supporting areas and the occlusal contact of the anterior teeth: group A had four functional occlusal supporting areas; group B had 1–3 functional occlusion areas, or no area with occlusal contact of incisors, lateral incisors or canines; group C had no occlusal supporting area with no occlusal contact of incisors, lateral incisors or canines (Eichner, 1990). Groups A/B/C represent good/moderate/poor occlusal support, respectively, Ikebe et al. (2010b).

### 2.3. Cognitive function assessment

A series of neuropsychological tests were used to assess the participants’ cognitive function in this study. Based on Chinese culture, we translated, adapted, and validated neuropsychological tests from Western countries. Because some tests require vocabulary, writing, or reading skills, we designed sets of neuropsychological tests based on the educational level of the participants. These tests included assessments of overall cognition (Zhang et al., 1990), command execution, spatial imagery, memory, language, and attention (Ding et al., 2015). The tests included: (1) the Mini-Mental State Examination (MMSE); (2) the Conflicting Instructions Task (Go/No Go Task); (3) the Stick Test; (4) the Modified Common Objects Sorting Test; (5) the Auditory Verbal Learning Test; (6) the Modified Fuld Object Memory Evaluation; (7) the Trail-making test A&B; (8) the Renminbi (official currency of China) Test, translated from the EURO test. The tests were completed within 90 min.

Participants were diagnosed with dementia according to DSM-IV criteria (Diagnostic and Statistical Manual of Mental Disorders, Fourth Edition) (Ding et al., 2014) and MCI according to Peterson’s criteria (Petersen, 2010). A detailed procedure for consensus diagnosis has been published previously (Ding et al., 2015).

### 2.4. Data collection of other variables

Trained investigators conducted face-to-face interviews with participants to collect their demographic characteristics (age, sex, height, weight, years of education, etc.) lifestyle habits (smoking, alcohol drinking), and medical histories of diabetes mellitus and cardiovascular diseases. Smoking and alcohol drinking habits mean smoking cigarettes and drinking alcohol for at least 1 year. BMI (Body Mass Index) is a person’s weight in kilograms divided by the square of height in meters. Medical history included a history of cardiovascular disease and diabetes mellitus, confirmed consistently by the investigators after checking the medical records.

### 2.5. Statistical analysis

For the current data, rates of Eichner classification group C participants with cognitive impairment and normal cognition were 0.8106 and 0.1894. Due to the sample size of 1,225, the power of the test (1-β) exceeded 99% with α = 0.05, according to the below formulation. Therefore the sample size was sufficient for our study.


$$n=\frac{Z_{\alpha /2}\,\sqrt{2\,p\,\left(1-p\right)}+Z_{\beta }\,\sqrt{{\text{p}}_{0}\,\left(1-{\text{p}}_{0}\right)+{\text{p}}_{1}\,\left(1-{\text{p}}_{1}\right)}}{{\left({\text{p}}_{1}-{\text{p}}_{0}\right)}^{2}}$$

Continuous variables were described in mean (standard deviation, SD) or median (interquartile range, IQR) when appropriate, and frequencies (%) were used for categorical variables. We used the Student *t*-test, Pearson Chi-squared test, and analysis of variance (ANOVA) to compare the variables. The association between the number of occlusal supporting areas/Eichner index and cognitive impairment was examined by logistic regression models. The number of occlusal supporting areas was treated as a continuous variable, while the Eichner index as an ordinal categorical variable. Model 1 was univariate, and Models 2 and 3 were multivariable models. Model 2 was adjusted for age and sex; Model 3 was adjusted for confounders, including age, sex, education duration, cigarette smoking, alcohol drinking, cardiovascular disease, and diabetes. In the model assessing the association between the Eichner index and cognitive impairment, group A was the reference group.

We used mediation analysis for the mediation effect of age between the number of functional occlusal supporting areas and cognitive impairment. We defined three pathways in the mediation analysis: (1) number of functional occlusal supporting areas to age, (2) age to cognitive impairment (direct effect) and (3) number of functional occlusal supporting areas to cognitive impairment (total effect). The total effect is calculated through the sum of the direct and mediation (indirect) effects. The percentage of the mediation effect is calculated using the following formula (mediation effect/total effect × 100%).

The *P*-values and 95% CIs were estimated in a two-tailed manner, and *P* < 0.05 was considered significant. Data were analyzed using SAS 9.4 (SAS Institute Inc., Cary, NC, USA).

## 3. Results

### 3.1. Baseline characteristics

The baseline characteristics of 1,225 participants were shown in Table 1. There were 660 patients with cognitive impairment (188 dementia and 472 MCI), accounting for 53.88% of the total population. The mean and standard deviation of age was 79.92(10.01), with BMI 22.92(3.67). Female participants accounted for 59.10%, slightly more than male ones. More than 60% of the participants received junior high school education or below. There were 12.73% of smokers and 5.47% of alcohol drinkers. They had an average number of 14.74 remaining teeth and 1.45 functional occlusal supporting areas. Age, sex, education, BMI, smoking, alcohol drinking, cardiovascular disease, diabetes mellitus, number of remaining teeth, number of functional occlusal supporting areas, Eichner classifications were significantly different between groups with cognitive normal and cognitive impairment (*P* < 0.05).

**TABLE 1:** Demographics of participants with or without cognitive impairment.

	All, *n* = 1225	Cognitive normal, *n* = 565	Cognitive impairment, *n* = 660	*P*-value
Sex				0.0009
Male, n (%)	501(40.90)	260(46.02)	241(53.98)	
Female, n (%)	724(59.10)	305(53.98)	419(46.02)	
Age, years, mean ± SD	79.92 ± 10.01	71.44 ± 6.50	87.18 ± 5.95	<0.0001
Education, n (%)				<0.0001
Primary school and below, n (%)	325(26.53)	44(7.79)	281(92.21)	
Junior high school, n (%)	428(34.94)	240(42.48)	188(57.52)	
Senior high school, n (%)	416(33.96)	255(45.13)	161(54.87)	
Undergraduates and above, n (%)	56(4.57)	26(4.60)	30(95.4)	
BMI, kg/m^2^, mean ± SD	22.92 ± 3.67	23.40 ± 3.46	22.50 ± 3.80	<0.0001
Cigarette smoking, n (%)	156(12.73)	108(19.12)	48(80.88)	<0.0001
Alcohol drinking, n (%)	67(5.47)	45(7.96)	22(92.04)	0.0006
Cardiovascular disease, n (%)	216(17.63)	62(10.97)	154(89.03)	<0.0001
Diabetes mellitus, n (%)	263(21.47)	104(18.41)	159(81.59)	0.0341
Remaining teeth, n, mean ± SD	14.74 ± 10.95	19.81 ± 10.00	10.40 ± 9.80	<0.0001
Functional occlusal supporting areas, n, mean ± SD	1.45 ± 1.34	2.07 ± 1.29	0.92 ± 1.15	<0.0001
Eichner classification, n (%)				<0.0001
Group A	67(5.47)	56(9.91)	11(90.09)	
Group B	705(57.55)	402(71.15)	303(28.85)	
Group C	453(36.98)	107(18.94)	346(81.06)	
Denture				<0.0001
Missing	620(50.61)	332(58.76)	288(43.64)	
Fixed partial denture	249(20.33)	106(18.76)	143(21.67)	
Removable partial denture	356(29.06)	127(22.48)	229(34.7)	
Stages of periodontitis, n (%)				<0.0001
Normal	0(0)	0(0)	0(0)	
Stage I	4(0.33)	4(0.71)	0(0)	
Stage II	46(3.76)	36(6.37)	10(1.52)	
Stage III	168(13.71)	128(22.65)	40(6.06)	
Stage IV	1,007(82.20)	397(70.27)	610(92.42)	

### 3.2. Association between occlusal support and cognitive impairment

**FIGURE 1:**
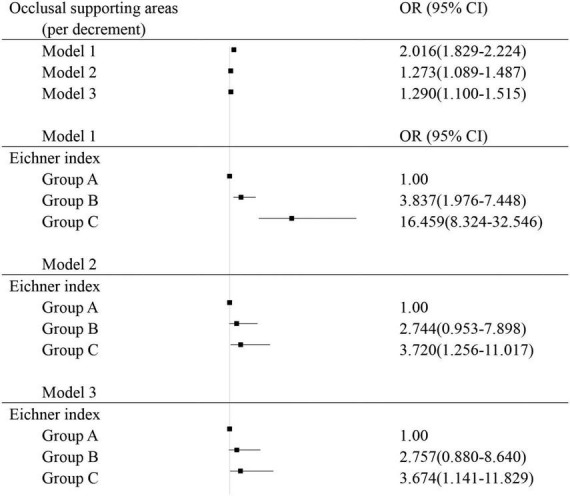
Logistic regression analysis for risk factors associated with cognitive impairment; Group A: 4 functional occlusal areas; Group B: 1–3 functional occlusal areas or 0 area with anterior occlusal contact; Group C: with no functional occlusal contact; Model 1: univariate; Model 2: adjusted for sex and age; Model 3: adjusted for age, sex, body mass index, education levels, smoking and alcohol drinking, diabetes, and cardiovascular disease.

The results of the association between the number of functional occlusal supporting areas and cognitive impairment using ordinal logistic regression models were shown in Figure 1. Model 1, a univariate model, showed that fewer functional occlusal supporting areas were associated with higher prevalence of cognitive impairment (OR = 2.016, 95%CI 1.829–2.224) compared to normal participants. Model 2 showed that fewer functional occlusal areas were associated with higher prevalence of cognitive impairment compared to normal participants (OR = 1.273, 95%CI 1.089–1.487). Model 3 showed that decreasing functional occlusal areas were associated with a higher likelihood of cognitive impairment compared to individuals with normal cognition (OR = 1.290, 95%CI 1.100–1.515) after adjusting for age, sex, years of education, smoking, alcohol drinking, cardiovascular disease, and diabetes.

The correlation between Eichner classifications and cognitive impairment was shown in Figure 1. Eichner classification was included as an ordered multi-categorical variable. Model 1, a univariate model, showed that compared to group A, group B had an OR of 3.837 for cognitive impairment (95%CI 1.976–7.448). Group C had an OR of 16.459 for cognitive impairment (95%CI 8.324–32.546). Model 2, adjusting for age and sex, showed that group C had an OR of 3.720 compared to group A (95%CI 1.256–11.017). After the adjustment of age, sex, education level, cigarette smoking, alcohol drinking, cardiovascular disease, and diabetes, model 3 showed that group C had an OR of 3.674 for cognitive impairment compared to group A (95% CI 1.141–11.829).

### 3.3. Mediation analysis of age

**FIGURE 2:**
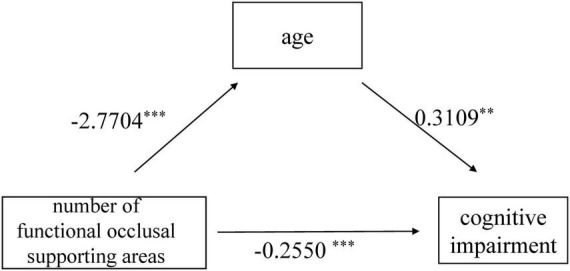
Mediation model of age as a mediator between number of functional occlusal supporting areas and cognitive impairment;^**^p < 0.01, ^***^p < 0.001.

The results of the mediation effects analysis suggested that the association between number of functional occlusal supporting areas and cognitive impairment was mediated by age after controlling the covariates including sex, education, cigarette smoking, alcohol drinking, diabetes, and cardiovascular disease (Table 2). When age was used as a mediator, a significant model was obtained (number of functional occlusal supporting areas → age, *p* < 0.001, β = −2.77,95% CI:−3.12,−2.42; age → cognitive impairment, *p* < 0.001, β = 0.31,95%CI: 0.27,0.35; number of functional occlusal supporting areas → cognitive impairment, *p* < 0.001, β = −0.26, 95%CI: −0.42, −0.09). Age mediated 66.53% of the association between number of functional occlusal supporting areas and cognitive impairment (β = 0.42, 95% CI: 0.36, 0.49) (Figure 2).

**TABLE 2:** Mediation analysis of age for the association between number of functional occlusal supporting areas and cognitive impairment.

Effect	Estimate	95% CI		*P*-value
Number of functional occlusal supporting areas →age	−2.77	−3.12	−2.42	*p* < 0.001
Age →cognitive impairment	0.31	0.27	0.35	*p* < 0.001
Number of functional occlusal supporting areas →cognitive impairment	−0.26	−0.42	−0.09	*p* < 0.001
Total effect	−0.67	−0.74	−0.60	*p* < 0.001
Direct effect	−0.23	−0.35	−0.10	*p* < 0.001
Mediated effect	−0.45	−0.54	−0.36	*p* < 0.001

Controlling for sex, education, cigarette smoking, alcohol drinking, diabetes mellitus, and cardiovascular disease.

## 4. Discussion

In the current cross-sectional study, we used functional occlusal support areas and the Eichner index to evaluate occlusal support. Our result indicated a significant association between lower occlusal support and a higher prevalence of cognitive impairment.

The present study found a significant correlation between cognitive impairment and the number of functional occlusal areas, consistent with previous studies (Miura et al., 2003; Weijenberg et al., 2011, 2019; Miquel et al., 2018; Nakamura et al., 2021). Masticatory dysfunction due to decreased occlusal support can contribute to chronic systemic diseases and is a risk factor for cognitive impairment (Shatenstein et al., 2012). Numerous studies have demonstrated the association between masticatory dysfunction and cognitive impairment (Paganini-Hill et al., 2012; Weijenberg et al., 2015, 2019). In a Leisure World cohort study among 5,468 old adults in the US, people with inadequate mastication wearing no dentures had an OR of 1.91 for dementia compared to those with adequate mastication (Paganini-Hill et al., 2012). Weijenberg et al. (2015) found significant positive associations between masticatory performance and general cognition in 114 old adults. Miura et al. (2003) investigated 88 elderly Japanese women, 44 of whom had normal cognitive function, and the other 44 had cognitive impairment. The masticatory function was evaluated using the number of remaining teeth, maximum bite force, and the number of occlusal functional areas. They found that masticatory function was significantly associated with cognitive impairment (Miura et al., 2003). Onozuka et al. (2003) used fMRI to find that chewing increased neuronal activity in some areas of the brain in a study of 32 participants. Lack of occlusal support, whether due to periodontal disease, tooth loss, or other factors, is associated with several other primary adverse health-related outcomes in older adults, such as frailty, hospitalization, falls, mortality, functional disability, and quality of life. Dibello et al. (2021, 2023) found a significant association between poor mastication and frailty.

However, some studies have found no significant or negative correlation between chewing and cognitive function. Tucha et al. (2010) found that chewing gum harmed cognitive function in children with Attention Deficit Hyperactivity Disorder (ADHD). Such negative results may be because the participants were young. The effect of chewing on cognitive function has now been found to be age-related, with its most potent effect on improving cerebral blood flow and cognitive function in older adults (Momose et al., 1997). Its potential long-term effects need to be further investigated.

As an unmodifiable risk factor, age is associated with multiple modifiable risk factors of cognitive impairment. As age increases, the number of teeth and occlusal function decreases. This study showed that age significantly mediated between occlusal support and cognitive impairment. It may be due to aging, which causes a decrease in blood flow and the promotion of neurodegeneration in the brain (Weijenberg et al., 2019). Chewing increased cerebral blood flow in primary sensorimotor areas by 25–28%, in supplementary motor and insular areas by 9–17%, and in the cerebellum and striatum by 8–11% (Momose et al., 1997). As it is well known that stress can impair cognitive function, chewing relieves stress in humans and allows them to maintain the same cognitive function under stress as in a relaxed state (Weijenberg et al., 2011). Studies in edentulous patients have shown that oral prosthetics can improve masticatory function, increase local cerebral blood flow, and prevent cognitive impairment (Miyamoto et al., 2005). Azuma et al. (2017) found that mastication helped to preserve hippocampus-related cognition through the hypothalamic-pituitary-adrenal (HPA) axis. Further studies were needed to determine the mechanism of chewing and cognitive impairment.

There were several limitations in this study. Firstly, the cross-sectional study showed the association between occlusal support and cognitive impairment but not the causal relationship. Secondly, we used the Eichner classifications as the indicator of occlusal support. Eichner classifications lack precise occlusal force values compared to other methods, including color-changeable gum chewing and masticatory force measurement. However, it is easy to implement and suitable for extensive sample-size studies. Thirdly, although we included many confounders, such as age and sex, some potential relevant factors may still be missing. Fourthly, we recruited community-dwelling older adults in the study, and it may be inappropriate to generalize our findings to other populations. Further cohort and *in vivo* studies are needed for the causal relationship and underlying mechanisms between occlusal support and cognitive impairment.

## 5. Conclusion

In conclusion, cognitive impairment was significantly associated with the number of missing teeth, functional occlusal areas, and Eichner classifications in this cohort with older community residents. Oral health should concern patients and doctors caring about cognitive impairment, especially occlusal support.

## Data availability statement

The raw data supporting the conclusions of this article will be made available by the authors, without undue reservation.

## Ethics statement

The studies involving human participants were reviewed and approved by the Medical Ethics Committee of Huashan Hospital Affiliated to Fudan University. The patients/participants provided their written informed consent to participate in this study.

## Author contributions

YZ and DDa conceptualized the work and approved all the protocol. DDa, SG, HZ, XZ, YJ, JY, HW, WW, XZ, XL, QZ, and DDing collected the data. DDa undertaken the statistical analysis. DDa and DDing prepared the manuscript. YZ and DDing are the guarantors of this manuscript. All authors contributed to the article and approved the submitted version.
